# Comprehensive observations and multidisciplinary approaches (COMA) in the management of unconscious patients: a prospective high fidelity simulation study

**DOI:** 10.1007/s00415-025-13228-4

**Published:** 2025-07-25

**Authors:** Nüesch Liliane, Kai Tisljar, Sebastian Berger, Gian Marco De Marchis, Tolga D. Dittrich, Stefano Bassetti, Roland Bingisser, Sabina Hunziker, Stephan Marsch, Raoul Sutter

**Affiliations:** 1 Intensive Care Unit, Department of Acute Medicine, University Hospital Basel, Petersgraben 4, 4031 Basel, Switzerland; 2 Neurology, University Research and Teaching Hospital, Kantonsspital St. Gallen, St. Gallen, Switzerland; 3 Medical Faculty, University of Basel, Basel, Switzerland; 4 Division of Internal Medicine, University Hospital Basel, Basel, Switzerland; 5 Department of Emergency Medicine, University Hospital Basel, Basel, Switzerland; 6 Department of Psychosomatic Medicine, University Hospital Basel, Basel, Switzerland

**Keywords:** Coma, Neurocritical care, Simulator study, Prospective study

## Abstract

**Background:**

Managing patients with coma of unknown etiology presents a challenge requiring rapid, structured assessment. We aimed to examine how physicians from different specialties manage patients with coma of unknown etiology and adhere to recommendations in a highly standardized scenario.

**Methods:**

Prospective high-fidelity simulation study conducted at an academic simulation center involving 50 physicians from acute care (38%), internal medicine (36%), and neurology (26%). Participants were confronted with a standardized coma scenario. Performance was assessed for adherence to expert-recommended clinical assessments (primary endpoints) and timing of interventions, such as airway protection, oxygen administration, toxicological screening, and self-evaluation (secondary endpoints).

**Results:**

All participants recognized coma; 80% assessed the Glasgow Coma Scale, with 40% quantifying it correctly. 20% completed ABCDE assessments, with 66% performing head-to-toe examinations. Airway inspection was conducted by 89% of acute care physicians, 70% of neurologists, and 60% of internists. A median of 4 ancillary tests were ordered, mostly neuroimaging (98%) and toxicological screening (86%), while rare toxin screening (2%) and EEG (12%) were scarce. Oxygen was universally administered (100%), but treatment response was rarely checked (8%). Side-positioning for airway protection was infrequent (21% acute care, 15% neurology, 6% internal medicine), while intubation was more commonly ordered by internists (17%). Prior simulator training improved side-positioning rates (27% vs. 4%, *p* = 0.047). Self-evaluations showed high motivation (median 8/10) but moderate confidence (5/10).

**Conclusions:**

This study highlights specialty-specific differences, misconceptions, and gaps in managing coma of unknown etiology, including inconsistent diagnostic workup and missed treatments, emphasizing the need for guidelines, standardized care and training.

**Registration:**

ClinicalTrials.gov registry (ID NCT06265168).

**Supplementary Information:**

The online version contains supplementary material available at 10.1007/s00415-025-13228-4.

## Introduction

Coma of unknown etiology is a neurological emergency frequently encountered in emergency settings but remains underinvestigated. Defined as acute consciousness impairment without overt causes, such as trauma or hypoperfusion [1], its incidence ranges from 0.4 to 2%, while altered mental status, a broader category, affects 5–9% of emergencies [1, 2]. Coma is characterized by the absence of wakefulness, awareness, and external responses, reflecting brain dysfunction with reversible or irreversible causes [3, 4]. Rapid diagnosis and treatment of reversible causes are crucial to prevent morbidity and mortality, which can reach up to 25% [1, 5]. Cases with unidentified causes present diagnostic challenges.

Although many centers have implemented standard operating procedures (SOPs) for managing unresponsive patients, universally accepted guidelines are lacking. Current approaches rely heavily on expert opinions [3, 6] rather than robust evidence and often omit or underemphasize internationally recommended steps like the ABCDE approach [7, 8]. The latter is a structured method for assessing and managing critically ill or injured patients prioritizing immediate life-threatening conditions thereby focusing on **a**irway, **b**reathing, **c**irculation, **d**isability and **e**xposure [7, 8].

To give more insight into the current quality of diagnostic procedures and treatment of patients with coma of unknown cause, this prospective high-fidelity simulation study was performed to examine how physicians from different specialties manage adult patients with coma of unknown etiology and adhere to current recommendations [3, 6], including the ABCDE approach, in a highly standardized simulated scenario [7, 8].

## Methods

### Ethical approval, registration, and consents

The study was approved by the ethics committee (EKNZ No.2023-01512) and registered on ClinicalTrials.gov (ID NCT06265168). Written informed consent was obtained from all participants.

### Setting and design

This prospective, investigator-led, single-blinded high-fidelity simulation study was conducted from March to November 2024 at the University Hospital Basel’s ICU-affiliated simulation center. While institutional SOPs for altered consciousness management were available, SOPs for coma of unknown etiology were not established. Although the established simulated scenario was primary used to investigate the quality of performance of physicians at our medical center, simulation workshops were formally offered for training purposes on emergency management to physicians in intensive care, emergency medicine, internal medicine, and neurology. Before the workshops, the participants had no prior training in the context of the simulated scenario. Voluntary participation was conducted during working hours without additional compensation. All eligible physicians completed a pre-simulation questionnaire on demographics, clinical knowledge, prior simulation experience, total clinical exposure, and pre-session workload.

The extended STROBE (**ST**rengthening the **R**eporting of OBservational studies in **E**pidemiology)-guidelines for simulation research were followed [9].

### Simulator setup

Information about the high-fidelity simulator center’s equipment is available in our prior studies [10–12]. Briefly, a programmable high-fidelity mannequin (SimMan^®^, Laerdal Medical AS, Stavanger, Norway) was utilized, capable of simulating various medical conditions. The mannequin could speak, groan, cough, exhibit palpable or absent pulses, thoracic movements, and various sounds of breathing, as well as blinking, pupil responses, upper extremity motion, foamy sputum production, and enuresis. Vital signs (respiratory rate, oxygen saturation, heart rate, and non-invasive blood pressure) were displayed on bedside monitors if measured by the participants and adjusted based on interventions by the participants. Emergency medications (vasopressors, antimicrobials, steroids, thiamine, fluids, glucose, and antiseizure drugs) were available alongside intubation equipment, and flashlights. During simulations, a trained nurse, as part of the simulated scenario, assisted by following physician instructions for diagnostics and monitoring device setup, maintaining a cooperative but passive demeanor.

### Simulated clinical scenario

The content validity of the simulated clinical scenario was based upon the following approach: first, the simulated scenario was based upon a well-established, studied and published previous scenario of a comatose adult patient presenting with a minimal convulsive status epilepticus [10, 12] by removing the motor symptoms (i.e., convulsions) and signs of enuresis. In addition, the accompanying laboratory handouts, which were provided to the participants, were adapted to fit the current scenario of intoxication. Second, the experienced study/simulator team consisting of a leading senior physician board certified in neurology and intensive care medicine, a first-year resident in her last year prior to graduation, and two experienced registered nurses qualified for intensive care medicine, generated and assessed the adapted scenario for content validity, until overall consensus was reached. The final scenario that was agreed upon was then piloted on two volunteering physicians who both recognized the intended scenario of coma. Third, our finding that in the study 100% of all participants recognized coma supports the content validity of our simulated scenario.

Prior simulation all participants were instructed to assume the role of the emergency department physician. Participants were briefed on the mannequin’s capabilities, including motion, vital sign displays, airway management, and repositioning. For their training, participants were confronted with a highly standardized simulation clinical scenario in an emergency room involving an adult comatose patient with an initial Glasgow Coma Score (GCS) of 3, which gradually improved to 4 (eye1, verbal2, motor1). Participants were blinded to the underlying cause of coma, which was an isopropanol intoxication. This rare cause of coma was selected to assure that participating physicians would rarely determine the underlying etiology of the coma early within the designated 20-min training period.

The scenario began with the participating physician being called by the nurse to assess a patient “not being well”. The simulated patient was positioned supine, exhibited a GCS of 3 and coughed repetitively. All vital signs were simulated within normal physiological ranges (if monitored by the participants), except for periodic oxygen desaturation slightly below 90%. The mannequin featured a pre-installed intravenous access. A printed medical chart included an emergency medical service report describing an adult patient with coma of unwitnessed onset and laboratory findings including a hemogram with normal leukocyte, thrombocyte, and erythrocyte counts, although erythrocytes were macrocytic. The C-reactive protein concentration, glucose levels, and thyroid-stimulating hormone concentrations were within normal limits, as were the transaminases. Alkaline phosphatase and total γ-glutamyltransferase, were mildly elevated. An unremarkable cerebral computed tomography (CT) was displayed at all times. Upon request, CT angiography was reported to be unremarkable. Upon request, blood gas analysis, conducted early in the clinical course, showed no significant acid–base disturbance, with normal pH and base excess, bicarbonate levels and electrolytes (consistent with findings in isopropanol intoxication [13]). Cerebrospinal fluid analysis revealed normal cell counts, lactate, and glucose. Albumin concentration was slightly reduced, while other proteins showed normal levels. Toxicological screening was negative for serum ethanol, benzodiazepines, barbiturates, opioids, methadone, cannabinoids, amphetamines, tricyclic antidepressants, acetaminophen, and salicylates and was reported upon request. Tests for less common toxins were pending. Upon request, an increased osmolal gap of 23 mOsm/kg H_2_O was presented.

The simulation was recorded via camera from different angles and a microphone for real-time observation, debriefing, and anonymized analysis. Sessions ended after 20 min or upon correct identification of isopropanol intoxication. Group debriefings followed each session.

After the simulation, participants completed two questionnaires (Supplemental File 1). The first assessed knowledge of coma diagnostics, management, and understanding. The second involved self-assessment using an “emotion wheel reflectivity” tool.

### Data assessment

And overview of all variables which were a priori identified, based upon a review by two of the authors (L.N. and R.S.) of the two references/guidelines regarding the assessment and management of coma [3, 6] and the two guidelines regarding the internationally recommended ABCDE approach [7, 8] that were systematically captured are presented in the Supplemental File 1. Participants’ performances and the “patient’s” vital signs (if measured and displayed on the monitor) were recorded simultaneously using “frame-in-frame” video and audio technology. Two independent observers (L.N. and R.S.) analyzed the recordings to code data for assessing the primary and secondary endpoints outlined below. Actions and verbalizations were coded on a “second-by-second” basis.

Inter-rater agreement on categorical variables was evaluated using Cohen’s kappa (*κ*), while continuous variables (e.g., time to action) were compared directly. Disagreements were resolved through joint review until consensus was reached.

### Outcomes and measurements

Primary endpoints included the proportion of participants who executed clinical assessments in line with recommended assessments for the management of coma [3, 6] including the internationally recommended ABCDE approach [7, 8] and the time of these executions after first “patient” contact.

Secondary endpoints included the performance and timing of medical interventions following initial assessments, such as airway protection (non-invasive side-positioning or tracheal intubation), oxygen administration, expanding toxicological screening to identify the etiology, and post-simulation self-evaluation.

### A priori sample size calculations

In the absence of established international guidelines for the management of patients with coma of unknown etiology, sample size calculation was based on data from comparable clinical scenarios involving similar treatment approaches, such as status epilepticus. Using a systematic review on adherence to status epilepticus guidelines [14], the hypothesized adherence rate was set at 80%. For a one-sample comparison against an observed proportion of 60% adherence rate to guidelines, the estimated required sample size was 43 participants assuming a one-sided significance level of 0.05 and a statistical power of 0.8.

### Statistics

For descriptive statistics, discrete variables were expressed as counts (percentage) and continuous variables were expressed as medians and interquartile ranges (IQR). Inter-rater agreement on categorical variables was evaluated using Cohen’s kappa (κ). Univariable comparisons of proportions were performed using chi-square test. Two-sided *p* values ≤ 0.05 were considered significant. Statistical analyses were performed with STATA^®^ version 16.1 (Stata Corp., College Station, TX, USA).

## Results

### Description of participants and inter-rater agreement

Demographics and baseline characteristics of the 50 participating physicians are presented in Table 1. Physicians were primarily affiliated with intensive care/emergency medicine (i.e., acute care specialties; 38%), internal medicine (36%), and neurology (26%). The median age was 31 years (IQR 29–33), with 64% being female. Participants had a median of 4 years of clinical experience (IQR 3–5), and 44% had prior simulation training. Males were slightly under-represented, with the representation being consistent with the current percentage of males in medical education in Switzerland (BAG Statistiken Ärztinnen/Ärzte, 2019). The working hours stated agreed with Swiss labor law.

**Table 1:** Baseline characteristics of participating physicians (*n* = 50)

Characteristics of participants	*n*/median	%/IQR
Age (years; median, IQR)	31	29–33
Female (*n*, %)	32	64.0
Physicians’ primary affiliation		
Intensive care/emergency medicine (*n*, %)	19	38.0
Internal medicine (*n*, %)	18	36.0
Neurology (*n*, %)	13	26.0
Years of clinical experience (years; median, IQR)	4	3–5
Previous simulator training (*n*, %)	22	44.0
Working hours prior to participation (hours; median, IQR)	10	8–11

*IQR* interquartile range

Inter-rater agreement regarding categorical variables assessed from video and audio recordings was *κ* = 0.93.

### Primary endpoints

**Fig. 1:**
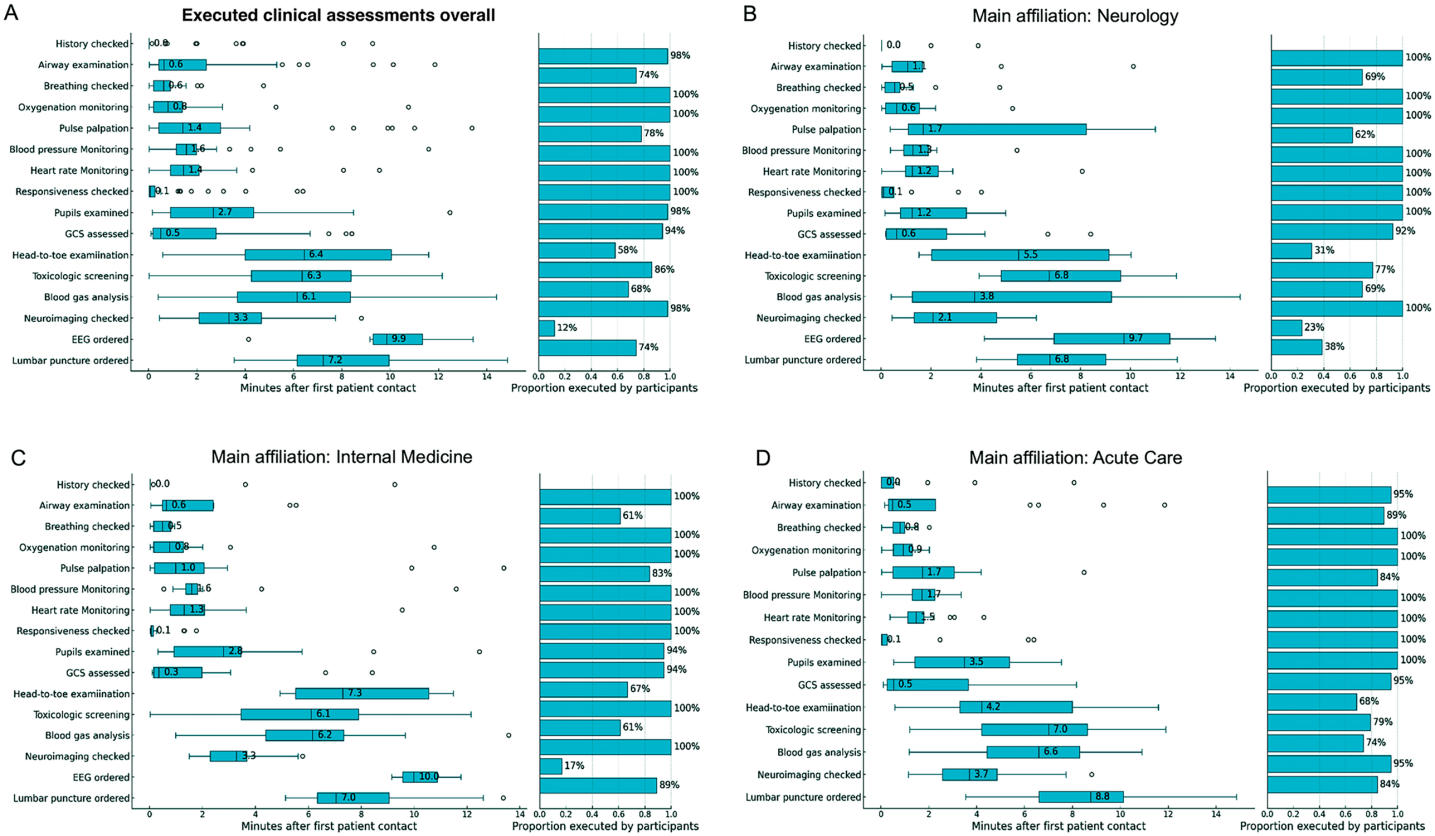
Proportion and time of executed clinical assessments for coma as recommended by opinion leaders [3, 6] including the internationally recommended ABCDE approach for the examination of critically ill patients [7, 8] categorized by the participants’ main affiliation (primary endpoints). *GCS* Glasgow Coma Score, *EEG* electroencephalography. *Note that boxplots represent attempted tasks without claiming correctness or completeness of task performance

The performance of clinical assessment is presented in Table 2. While the “patient’s” history and responsiveness were checked by almost all participants and coma was recognized by 100%, the level of consciousness was quantified correctly in 40% and complete execution of all ABCDE examination steps was performed in 20%. Important steps, such as airway inspection, pulse palpation, and a complete head-to-toe examination, were performed in 60–78%. While a median of four ancillary tests were performed/checked (IQR 3–4) with 98% checking neuroimaging for structural pathologies and 86% calling for toxicological screening results, other ancillary tests, including EEG, cerebrospinal fluid analyses were infrequently performed/checked or considered. Only 2% of the participants requested screening for rare toxins and none sought to calculate an osmolal gap, despite 86% having reviewed the general toxicology screening and 68% having analyzed the blood gas results. Figure 1A presents details regarding the overall percentage and time of executed examination steps after first “patient” contact. Assessments (if considered) such as head-to-toe examination, laboratory analyses, initiation of an EEG, and lumbar puncture were performed or considered with a median latency exceeding 6 min, in contrast to other assessments that were completed within 3 min. The proportion of executed assessments and time to performance after first patient contact categorized by the participants’ main affiliations is presented in Fig. 1B to D. Affiliation-centered subgroup analyses revealed a similar proportion of performed clinical assessments among all different specialties. However, while neurologists and internal medicine specialists performed airway examination in 60–70%, acute care specialists examined the airways more frequently (89%).

**Table 2:** Overall clinical performances and conceptual understanding of coma of the participating physicians (*n* = 50)

Overall clinical performance		
*Overall execution of first assessment (primary endpoints; n, %)*^a^		
Patient’s history assessed	49	98.0
Coma recognized	50	100.0
Responsiveness checked	50	100.0
GCS scored completely at least once	40	80.0
GCS scored correctly	20	40.0
Call for additional staff	46	92.0
All ABCDE systems checked at least once	10	20.0
**A**irway checked at least once	37	74.0
**B**reathing checked at least once	50	100.0
**C**irculation/pulse checked at least once	39	78.0
**D**isability (neurologic) checked at least once	50	100.0
**E**xposure/head-to-toe examination at least once	33	66.0
*Critical ancillary tests (secondary endpoints)*^a^		
Number of ancillary tests checked/performed at least once (median, IQR)	4	3–4
Toxicological screening checked at least once (*n*, %)	43	86.0
Call for extended toxicological screening for rare toxins at least once (*n*, %)	4	2.0
Calculation of osmolal gap at least once (*n*, %)	0	0.0
Blood gas analysis checked at least once (*n*, %)	34	68.0
Neuroimaging checked at least once (*n*, %)	49	98.0
EEG requested at least once	6	12.0
Lumbar puncture ordered and CSF analysis checked at least once	37	74.0
*Overall executed treatment steps (secondary endpoints; n, %)*^a^		
Side positioning for airway protection	7	14.0
Call for tracheal intubation	6	12.0
Oxygen supply	50	100.0
Antiseizure drugs administered	7	14.0
Antibiotics administered	1	2.0
Antiviral drugs administered	1	2.0
Treatment response checked	4	8.0
*Conceptual statements regarding coma (secondary endpoints as assessed by questionnaires; n, %)*		
Coma definition correct	28	56.0
Suspected etiology “intoxication” to be most likely	46	92.0
Indicating that “intoxication” is a frequent and important etiology	18	36.0
Considering seizures as the cause of coma	32	64.0
Considering cerebral hemorrhage as the cause of coma	47	94.0
Considering traumatic brain injury as the cause of coma	45	90.0
Considering aspiration with hypoxemia as the cause of coma	16	32.0
Indicating that comatose patients must be intubation regardless of underlying cause	11	22.0

*GCS* Glasgow Coma Score (range 3–15) [25, 26], *IQR* interquartile range, *EEG* electroencephalography, *CSF* cerebrospinal fluid

**Fig. 2:**
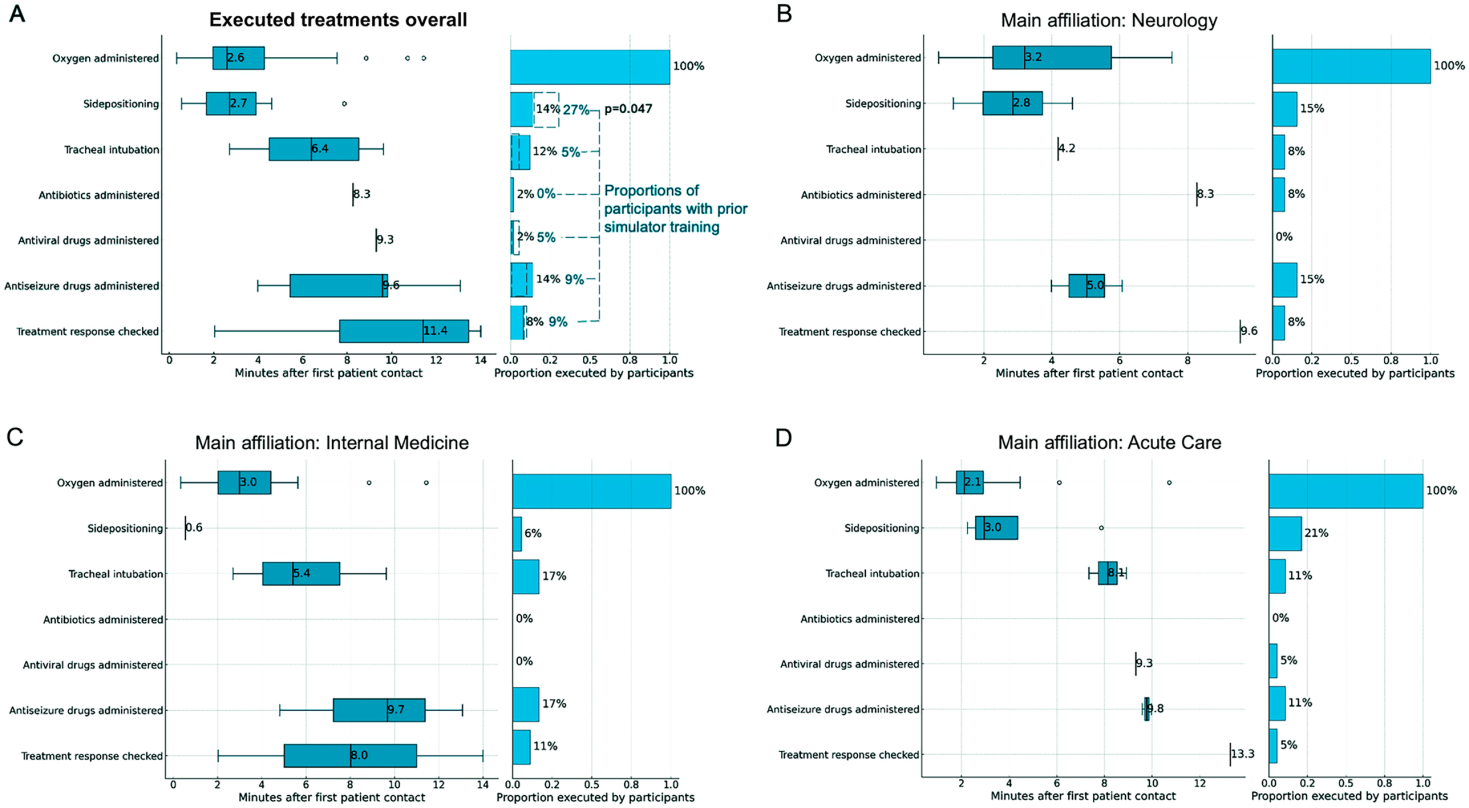
Proportion and time of executed treatment steps for coma as recommended by opinion leaders [3, 6] categorized by the participants’ main affiliation (secondary endpoints)

^a^More detailed analyses are presented in Figs. 1 and 2

### Secondary endpoints

Figure 2A illustrates the proportion of participants initiating treatments and the timing of various treatment steps following initial “patient” contact. While oxygen administration was universally performed, airway protective measures such as side-positioning and tracheal intubation were less frequently executed (14% and 12%, respectively). Antibiotic and antiviral therapies were initiated by two participants, whereas antiseizure medication was more commonly administered (14%). Treatment response evaluation was rarely undertaken, occurring in 8% of scenarios.

Analyses of treatment initiation and timing, stratified by the physicians’ specialty, are presented in Fig. 2B to D revealing consistent performance distributions across specialties, with few notable exceptions. Internal medicine specialists performed side-positioning significantly less frequently than neurologists (6% vs. 15%) and acute care specialists (6% vs. 21%). Conversely, tracheal intubation was requested more often by internal medicine specialists compared to neurologists (17% vs. 8%). Participants with prior simulator training of emergency scenarios performing side-positioning more often as compared to untrained participants (27% vs. 4%; *p* = 0.047; Fig. 2A).

### Concept of coma and self-evaluation (secondary endpoints)

First questionnaires (Supplemental File 2) revealed several conceptual misunderstandings, as detailed at the bottom of Table 2. Nearly half of the participants provided incorrect definitions of coma. In addition, treatment misconceptions were identified, with over 20% of physicians asserting immediate tracheal intubation to be mandatory for comatose patients, irrespective of underlying etiologies.

**Fig. 3:**
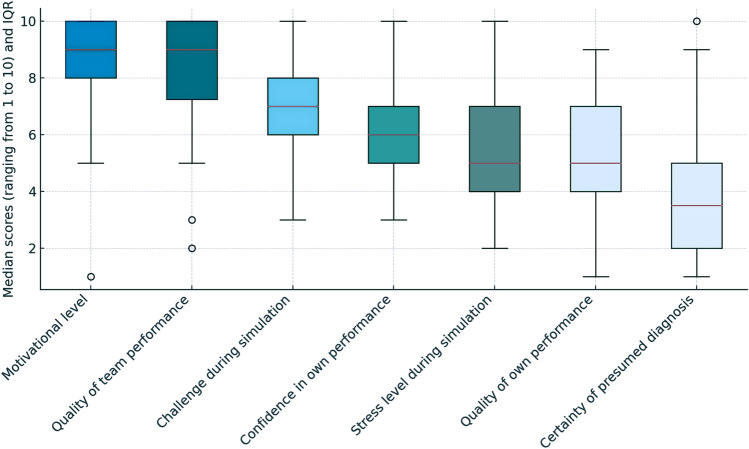
Debriefing with self-evaluation of performance during simulation (secondary endpoints)*. *IQR* interquartile range. *Ranges for self-evaluation according to the emotional reflexivity questionnaire with “1” indicating “not at all” or “very uncertain” and “10” indicating “very much”, “excellent” or “very certain”

The results of the self-evaluation (Supplemental File 2) are presented in Fig. 3. While most physicians reported high motivation and rated the quality of team performance as high, their median self-assessment of personal challenge and confidence during the scenarios ranged from moderate to high. In contrast, the quality of their own performance and the perceived stress level were mostly rated as moderate. Furthermore, their confidence in the accuracy of their presumed diagnosis was reported as low to moderate.

## Discussion

This study examined how physicians from various specialties manage an adult patient with coma of unknown etiology in a highly standardized high-fidelity simulation, assessing adherence to recommended measures [3, 6] (primary endpoints), as well as the timing and execution of medical interventions, and post-simulation self-evaluation (secondary endpoints). The findings reveal significant variability in diagnostic and therapeutic practices and highlight important and critical gaps in adherence to recommended assessment bundles and treatment. Despite a 100% recognition of coma, only 40% of physicians quantified the level of consciousness accurately. Physicians completed the ABCDE bundle [7, 8] in only 20%. Crucial diagnostics, such as airway inspection, routine laboratory analyses, and advanced investigations, such as EEG, lumbar puncture, or extended toxicological screening, were frequently omitted. Treatment initiations varied markedly, with a concerningly high omission rate of essential steps. While oxygen was administered universally, airway protective measures, such as side-positioning or tracheal intubation, were notably infrequent, increasing the risk of aspiration and pneumonia, which can exacerbate systemic inflammation, worsen brain dysfunction [15], and prolong treatment and hospital stays [16].

Although our analyses revealed relatively consistent performance across specialties, acute care specialists conducted airway examinations more frequently than internal medicine physicians and neurologists. Internal medicine specialists were less likely to perform side-positioning but more often opted for tracheal intubation. This suggests specialty-specific variations that may reflect differences in training emphasis and clinical experience.

Conceptual gaps in understanding coma were evident, as nearly half of the participants provided incorrect coma definitions. Misconceptions about treatment were prevalent with over 20% mistakenly stating that all comatose patients require immediate intubation regardless of the underlying cause of coma. While participants rated their motivation and teamwork highly, confidence in their own diagnostic accuracy and performance was moderate, reflecting challenges of managing such complex scenarios—potential mediators for perceived stress and uncertainty as indicated by the physicians.

The inconsistent adherence to recommended assessments, particularly regarding airway assessment and management as well as full-body examination to detect injuries, aligns with previous simulation-based studies. A study evaluating lifeguards’ capacity to perform the ABCDE approach on a simulated drowned victim found that none of the participants performed complete clinical assessments [17]. Similarly, investigations in a simulated scenario of patients in status epilepticus revealed complete ABCDE assessment in only 5% [10, 12]. The absence of “head-to-toe” examinations in our scenario is another concern, given the likelihood of falls and injuries in unwitnessed coma cases, as noted in the simulated medical report. The mannequin’s design, however, may represent a bias, as it may have hindered such assessments. Nevertheless, these findings collectively highlight a pervasive challenge in the consistent application of systematic assessment protocols across various emergency situations, calling for more structured training and stronger emphasis of more systematic assessments and the implementation of guidelines.

This study further highlights the influence of prior simulator-based training, particularly in the areas of airway management and evaluating treatment responses with pre-trained physicians performing these steps more frequently. The limited number of participants with prior simulation training, however, calls for larger-scale research to fully understand its impact on performance. Nonetheless, our findings align with evidence that regular training in standardized emergency procedures improves outcomes and is a cornerstone of medical practice [18]. High-fidelity simulation has enabled clinicians across all experience levels to safely learn, refine, and repeatedly practice their skills without endangering patients or relying on high patient volumes. Therefore, training in simulated, high-pressure, time-sensitive neurologic emergencies, such as coma, may enhance the delivery of evidence-based care in real-world emergency settings.

Our previous findings in simulated scenarios of status epilepticus revealed significant differences among specific specialties [10, 12] motivated us to further investigate differences regarding coma assessment and management among different physicians’ specialties. This revealed very similar to our present study a less sufficient airway assessment and airway protective measures performed by internal medicine specialists and neurologists as compared to acute care specialists. We further enhanced our discussion by deducting that these observed disparities, especially regarding protective airway management indicate that implementing specialized training programs customized to distinct medical specialties may effectively mitigate these challenges. Furthermore, the findings underscore the potential benefit of supplementary interventions, such as instituting regular, standardized, and enhanced interdisciplinary debriefings following the management of coma patients. Such structured sessions would facilitate exposure to critical and constructive feedback, thereby fostering collaborative learning and knowledge exchange among physicians in daily clinical practice. The finding that physicians with prior simulation training performed side-positioning more frequently further underscores that more exposure to such scenarios may optimize clinicians performance and that less experienced physicians should have more interprofessional exchange, where they can profit and learn especially from more senior or experienced colleagues, an optimization that may be achieved through regular debriefing sessions that are still under established and rarely used in clinical practice [19].

The inverse relationship between affiliation to internal medicine or neurology and firm airway assessment/management warrants attention and emphasizes the need for stronger focus on this approach in guidelines. Despite coma of unknown etiology [5] and intoxication-related coma [20] is a common emergency faced by these specialists, no large studies have assessed the impact of simulation training on neurologists’ performance in managing coma. Simulation training for other neurological emergencies showed that participants found the scenarios realistic, reported improved medical knowledge, confidence in handling emergencies, and leadership skills [10, 12, 21–23]. The current simulator setup provides structured training and debriefing for managing coma of unknown etiology, aiming to establish a standard comparable to other advanced life support scenarios.

The inconsistent care of comatose patients with unknown causes reveals a critical need for standardized guidelines and training. To improve outcomes, structured training, interdisciplinary collaboration, and simulation-based education with feedback must be implemented. Future research should evaluate these interventions and refine protocols.

### Limitations

This study has several limitations. Its single-center design may restrict the generalizability of our findings. The simulation environment may also have limited external validity, necessitating confirmation of our findings in real-world clinical practice. Nonetheless, the fact that all participating physicians recognized patient unresponsiveness and that 94% attempted to quantify the level of consciousness suggests that the simulated scenario was realistic and effectively portrayed the intended neurological emergency. Moreover, several of our findings are consistent with those from clinical studies on neurological emergencies involving altered consciousness, such as epileptic seizures and status epilepticus, as previously investigated in simulation settings [10, 12]. In addition, high-fidelity simulation is widely employed as a training tool for advanced life support, providing effective learning opportunities for standardized and controlled clinical practice without posing risks to patients [24]. The inability to capture unspoken considerations of differential diagnoses, leaves it unclear whether certain diagnostic steps were omitted due to context-based exclusion of specific diagnoses. The fact that the video and audio material was reviewed by only two and not more reviewers represents another limitation, as well as the fact that both reviewers were not blinded to the participants clinical specialties which may be a source of bias. Finally, it is possible that some participants might have conducted additional examinations if the scenario would have been simulated for more than 20 min. However, we decided to use a 20 min time-window, as in an acute emergency, a delay in diagnosis over several minutes to hours can be critical and must be avoided.

## Conclusions

This high-fidelity simulation study reveals significant gaps in managing adult patients with coma of unknown etiology, including inconsistent diagnostics, incomplete adherence to expert recommendations, and missed critical treatments. Specialty-specific differences and misconceptions highlight the need for standardized care through guidelines and enhanced training. High-fidelity simulation offers a platform to improve practice, confidence, teamwork, and leadership. Structured training, guideline adherence, and inter-specialty collaboration are essential to improve diagnostic accuracy, treatment consistency, and patient outcomes, while targeted education and further research can refine protocols and elevate emergency care quality.

## Data availability statement

The corresponding author has access to all data. He takes full responsibility for the integrity of the data, the accuracy of the data analysis and interpretation, and the conduct of the research. The authors have the right to publish any and all data, separate and apart from the guidance of any sponsor.

## Supplementary Information

Below is the link to the electronic supplementary material.

Supplementary file1 (PDF 359 KB)

Supplemental File 1: Variables which were a priori identified and systematically captured, based upon a review of the four references/guidelines. (PDF 38 KB)

Supplemental File 2: Questionnaire regarding the past scenario and emotional reflexivity questionnaire for subjective self-evaluation after completing the simulated clinical scenario. (PDF 28 KB)

## Declarations

### Use of artificial intelligence-assisted technologies

The large language model (ChatGPT-4omni, OpenAI, San Francisco, USA) was used to paraphrase and summarize some of the manuscript’s content. After using this application, the authors further edited the content as needed and verified its accuracy.
