# Supplementary material for: Comprehensive observations and multidisciplinary approaches (COMA) in the management of unconscious patients: a prospective high fidelity simulation study
Source: J Neurol. 2025 Jul 25;272(8):537. doi: 10.1007/s00415-025-13228-4 (PMC12296809; doi:10.1007/s00415-025-13228-4)
Supplement: Supplementary file 2 — Supplemental File 1: Variables which were a priori identified and systematically captured, based upon a review of the four references/guidelines. (PDF 38 KB) [file 415_2025_13228_MOESM2_ESM.pdf]

Variables which were a priori identified and systematically captured, based upon a review of the four references/guidelines [1-4]

Overall clinical performance

Overall execution of first assessment (primary endpoints)

According to the recommendations of assessment and management of coma [1, 2]

- Patient’s history assessed
- Time from simulation start until patient’s history assessed
- Coma recognized
- Time from simulation start until coma recognized
- Responsiveness checked
- Time from simulation start until responsiveness checked
- GCS scored completely at least once
- Time from simulation start until GCS scored completely at least once
- GCS scored correctly
- Call for additional staff
- Time from simulation start until call for additional staff

According to the recommendations of the ABCDE approach for general em*ergency management [3, 4]*

- All ABCDE systems checked at least once
  - **A**irway checked at least once
  - Time from simulation start until airway checked at least once
  - **B**reathing checked at least once
  - Time from simulation start until breathing checked at least once
  - **C**irculation / pulse checked at least once
  - Time from simulation start until circulation / pulse checked at least once
  - **D**isability (neurologic) checked at least once
  - Time from simulation start until disability (neurologic) checked at least once
  - **E**xposure / head-to-toe examination at least once
  - Time from simulation start until exposure / head-to-toe examination at least once

Critical ancillary tests (secondary endpoints)

According to the recommendations of assessment and management of coma [1, 2]

- Number of ancillary tests checked/performed at least once
- Toxicological screening checked at least once
- Time from simulation start until toxicological screening checked at least once
- Call for extended toxicological screening for rare toxins at least once
- Time from simulation start until call for extended toxicological screening for rare toxins at least once
- Calculation of osmolal gap at least once
- Time from simulation start until calculation of osmolal gap at least once
- Blood gas analysis checked at least once
- Time from simulation start until blood gas analysis checked at least once
- Neuroimaging checked at least once
- Time from simulation start until neuroimaging checked at least once
- EEG requested at least once
- Time from simulation start until EEG requested at least once
- Lumbar puncture ordered and CSF analysis checked at least once
- Time from simulation start until lumbar puncture ordered and CSF analysis checked at least once

Overall executed treatment steps (secondary endpoints)

According to the recommendations of assessment and management of coma [1, 2]

- Side positioning for airway protection
- Time from simulation start until side positioning for airway protection
- Call for tracheal intubation
- Time from simulation start until call for tracheal intubation
- Oxygen supply

Time from simulation start until oxygen supply
Treatment response checked
Time from simulation start until treatment response checked

I addition, unrecommended but frequently performed actions were captured as recognized during video and audio review

References/guidelines

1. Traub SJ, Wijdicks EF (2016) Initial Diagnosis and Management of Coma. Emerg Med Clin North Am 34:777-793
2. Edlow JA, Rabinstein A, Traub SJ, Wijdicks EF (2014) Diagnosis of reversible causes of coma. Lancet 384:2064-2076
3. Soar J, Nolan JP, Bottiger BW, Perkins GD, Lott C, Carli P, Pellis T, Sandroni C, Skrifvars MB, Smith GB, Sunde K, Deakin CD, Adult advanced life support section C (2015) European Resuscitation Council Guidelines for Resuscitation 2015: Section 3. Adult advanced life support. Resuscitation 95:100-147
4. Truhlar A, Deakin CD, Soar J, Khalifa GE, Alfonzo A, Bierens JJ, Brattebo G, Brugger H, Dunning J, Hunyadi-Anticevic S, Koster RW, Lockey DJ, Lott C, Paal P, Perkins GD, Sandroni C, Thies KC, Zideman DA, Nolan JP, Cardiac arrest in special circumstances section C (2015) European Resuscitation Council Guidelines for Resuscitation 2015: Section 4. Cardiac arrest in special circumstances. Resuscitation 95:148-201
