# Supplementary material for: Comprehensive observations and multidisciplinary approaches (COMA) in the management of unconscious patients: a prospective high fidelity simulation study
Source: J Neurol. 2025 Jul 25;272(8):537. doi: 10.1007/s00415-025-13228-4 (PMC12296809; doi:10.1007/s00415-025-13228-4)
Supplement: Supplementary file 3 — Supplemental File 2: Questionnaire regarding the past scenario and emotional reflexivity questionnaire for subjective self-evaluation after completing the simulated clinical scenario. (PDF 28 KB) [file 415_2025_13228_MOESM3_ESM.pdf]

Questionnaire regarding the past scenario

What is the suspected diagnosis?...

What is the presumed etiology?...

Have you assessed a GCS (Glasgow Coma Scale)? □ YES □ NO

If YES, what was the GCS?..

Are you aware of any guidelines or opinion papers on the management of patients with initially unclear coma? □ YES □ NO

How is coma defined?..

What is the most common cause of coma?..

What is the first treatment measure for comatose patients?...

Is imaging always necessary for patients with initially unclear coma? □ YES □ NO

Which type of imaging is necessary?...

Should a patient with a GCS ≤ 8 be intubated? □ YES □ NO □ It depends

Simulation Feedback Questionnaire

How was your stress level during the simulation?

1 • 2 • 3 • 4 • 5 • 6 • 7 • 8 • 9 • 10

Not stressed Severely stressed

How was your motivation during the simulation?

1 • 2 • 3 • 4 • 5 • 6 • 7 • 8 • 9 • 10

Not motivated Strongly motivated

How challenging was the simulation?

1 • 2 • 3 • 4 • 5 • 6 • 7 • 8 • 9 • 10

Not at all Very much

How certain were you?

1 • 2 • 3 • 4 • 5 • 6 • 7 • 8 • 9 • 10

Uncertain Certain

How good was your clinical performance?

1 • 2 • 3 • 4 • 5 • 6 • 7 • 8 • 9 • 10

Not good Excellent

How good was the performance of the team?

1 • 2 • 3 • 4 • 5 • 6 • 7 • 8 • 9 • 10

Not good Excellent

How certain were you regarding the diagnosis?

1 • 2 • 3 • 4 • 5 • 6 • 7 • 8 • 9 • 10

Uncertain Certain
